# The Use of EZ Derm® in Partial-Thickness Burns: An Institutional Review of 157 Patients

**Published:** 2013-03-07

**Authors:** Jared Troy, Rachel Karlnoski, Katheryne Downes, Kimberly S. Brown, C. Wayne Cruse, David J. Smith, Wyatt G. Payne

**Affiliations:** *Division of Plastic Surgery and University of South Florida, Tampa; †Department of Research/Biostatistics, University of South Florida, Tampa; ‡Tampa General Regional Burn Unit, Tampa, FL; §Florida Gulf-to-Bay Anesthesiology, Tampa, FL; ∥Institute for Tissue Regeneration, Repair, and Rehabilitation, Surgical Service, Department of Veteran Affairs, Bay Pines VA Health System, Bay Pines, Fl

## Abstract

**Objectives:** To evaluate the use of EZ Derm® (Molnlycke Health Care, US, LLC, Norcross, GA) on partial-thickness burns. **Methods:** A retrospective review of medical records from patients presenting to the Tampa General Regional Burn Center from January 1, 2008, through January 1, 2012, was conducted. A hospitalwide list of patients was generated on the basis of the presence of charge codes for EZ Derm®. All encounters that did not pass through the Burn Unit were excluded. Applicable charts were reviewed for basic patient characteristics, burn characteristics, outcomes, and complications. Complications were defined as premature separation of EZ Derm®, deviation from a flat fully epithelized wound at the time of final EZ Derm® separation and hypertrophic/keloid scaring. **Results:** A total of 157 patients were identified and met the study criteria. Eighteen complications were reported from 16 of the 157 patients. Complications were attributed to positioning (2/133 = 1.5%), infection (4/133 = 3.0%), incomplete epithelialization at time of separation (3/133 = 2.2%), need for additional excision and grafting (6/133 = 4.5%), hypertrophic scaring (2/60 = 3.3), and cryptogenic (1/133 = 0.75). **Conclusions:** EZ Derm® has proven to be a robust wound dressing that provides cost-effective, consistent durable wound coverage with minimal complications that resolve without long-term sequela.

The use of biologic dressings has become an integral part of modern-day burn care. While allograft remains the standard by which all other dressing are compared, supply limitations, possible disease transmission, and cost limit its use.^1^^-^3 To overcome these limitations, efforts have been undertaken to seek alternative wound dressings.^2^^,^^4^ The ideal wound dressing would be inexpensive, nonantigenic, water permeable, and antibacterial; it will reduce pain, be adherent and flexible, provide protection to the wound, be easy to handle, and have a long shelf life. Many alternatives exist; however, porcine-derived products are commonly used biologic dressings because of how close they come to meeting the ideal criteria.^4^^-^6

EZ Derm® is an aldehyde cross-linked porcine dermis that has been available since the mid 1980s for the care of partial-thickness burns.^7^ It is an off-the-shelf, easy-to-handle dressing that has been shown to reduce pain, be cost-effective, and provide an environment conducive to wound healing.^6^ Despite its long-term availability and use, only early accounts of its use are reported in the literature, with only scarce accounts found more recently.^8^^,^^9^ In the Tampa General Regional Burn Unit, it has been used in the management of partial-thickness burns since 2008. Here, we present our experience with EZ Derm®, which represents the largest case series to date from a single institution to investigate short- and long-term outcomes of EZ Derm®.

## METHODS

After attaining approval of the University of South Florida institutional review board, retrospective data were obtained from the medical records of patients admitted to the burn unit and were treated with EZ Derm® from January 1, 2008, through January 1, 2012. All patients admitted to the tampa general hospital burn unit whose billing information included a charge code for EZ Derm® were included in the study. There were no exclusion criteria. Applicable charts were reviewed for basic patient characteristics, burn characteristics, outcomes, and complications. Complications were defined as premature separation of EZ Derm®, deviation from a flat fully epithelized wound at the time of final EZ Derm® separation and hypertrophic/keloid scaring.

For all patients, wound preparation began in the operating room with a Betadine preperation followed by sharp debridement to remove any dead or necrotic tissue. The wound was then assessed for its probability to heal spontaneously within 3 weeks (partial-thickness injury). Criteria for the use of EZ Derm® included nonvariable depth burns (only partial-thickness injuries); patients able to undergo operative surgical debridement/wound preparation (if necessary); burn unit admission (no nonadmission outpatient burn treatment); and adult patients. We did not utilize EZ Derm® on hand burns or pediatric patients. If the wound was deemed to be of acceptable depth (partial thickness), nonmeshed EZ Derm® was applied without suture or stapling fixation. When crossing a joint, the EZ Derm® was cut along the joint creases to prevent unnecessary restriction. It was not utilized on hands because of desire for continued motion and concern for shearing of the graft. All wrinkles and fluid collections were removed, and the grafts were secured with bandaging of dry Kerlix. Splints were applied when necessary to protect the EZ Derm® and allow time for adequate wound adherence.

On postoperative day (POD) 1, the Kerlix secondary dressing was removed and the EZ Derm® was left open to air. EZ Derm® was typically yellow, dry, stiff, and firmly adherent to the wound; areas of moisture were usually pale and white. Any hematomas or fluid collections were drained. If all wounds were covered and there was no anticipated need for further intervention, the patient was discharged with a follow-up visit scheduled for the following week. Patients were instructed to keep the EZ Derm® dry and to cut away the excess as the edges pealed up. Alternatively, if the patient had a combination of wounds treated by local wound care (primarily Silvadene dressing changes) in addition to EZ Derm®, they were discharged provided that they were reliable and able to perform adequate wound care at home. If the EZ Derm® was not dry on POD 1, heating lamps were used to facilitate the drying process.

On follow-up, any remaining EZ Derm® was inspected and trimmed as necessary. The wounds were monitored for signs of infection, hypertrophic scaring, failure to adhere, and incomplete healing. If EZ Derm® remained firmly adhered 2 to 3 weeks after the application, it was removed in the clinic by soaking with saline and beta glucan. After removal, the wound was reassessed for the need of additional excision and grafting.

## STATISTICAL ANALYSIS

Statistical analysis was conducted using SPSS 20.0 (IBM, Armonk, NY). Descriptive statistics are reported as frequencies and percentages for categorical variables and as means, medians, standard deviations, and ranges for continuous variables. Groups were compared using Fisher exact and chi-square tests (where appropriate) for categorical variables and Mann-Whitney *U* and *t* tests (where appropriate) for continuous variables. A *P* < 0.05 was considered statistically significant.

## RESULTS

**Figure 1:**
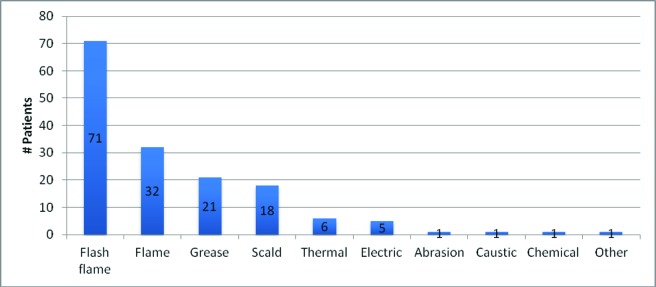
Mechanism of burn injury of patients for this study.

From January 1, 2008, through January 1, 2012, 157 patient records were identified. Table 1 illustrates basic patient demographics. Age ranged from 17 to 68 years (mean = 37.3 years). The subjects were predominantly male (80.9%), with an average body mass index = 26.8 (standrd deviation (STD) = 5.3). Burn total body surface area (TBSA) ranged from 1% to 80% (average = 16%, STD = 13.3), with flash flame (45.2%) and flame (20.4%) representing the majority of burn mechanisms followed by grease (13.4%) and scald (11.5%), Figure 1.

**Figure 2:**
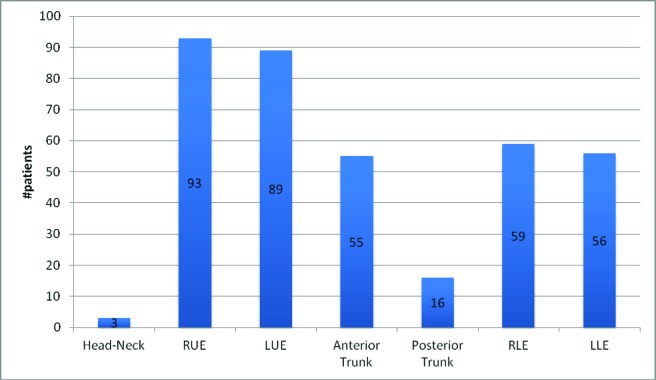
Treatment site.

The average size (length × width) of applied EZ Derm® was 2155 cm^2^ (STD = 1715 cm^2^, Median = 1750 cm^2^). The site of application was symmetric for upper and lower extremities, with a slight predisposition to the upper extremities (59% vs 37.3%, respectively). Application to the anterior trunk was more common than the posterior (35.7% vs 10.4%, respectively) and was rarely applied to the head/neck (1.9%), Figure 2.

**Figure 3:**
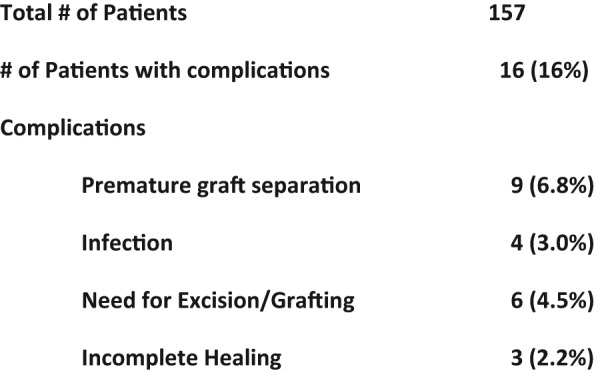
EZ Derm® complications.

The time from the initial burn injury until application of EZ Derm® was 4 days on average (STD = 2.8, Median = 3). The time from application to hospital discharge was 10 days on average (STD = 17.6, Median = 5). When subdivided into burn depth classification, an expected trend of increasing length of stay (LOS) is seen with increasing burn severity. On review of the data, it was noted that patients with documented inhalation injuries consistently had prolonged hospitalizations compared with patients without inhalational injury (median days: 28.5 vs 3, respectively; *P* < .001; Fig 3). A similar analysis was performed on overall LOS with an average of 15.4 days (STD = 27.8, Median = 36.5) and 8.9 days (STD = 7.9, Median = 7) for all patients with and without inhalation injuries, respectively, *P* < .001.

The average length of follow-up was 94.2 days (STD = 125.9, Median = 36). Of patients, 15.3% (24/157) were lost to follow up, and complete EZ Derm® separation and/or complete wound healing data could not be obtained. A comparison of these 2 groups revealed no significant difference in patient demographics.

A total of 18 complications were reported in 16 of the 157 patients as shown in Table 3. Complications consisted of premature separation, infection, incomplete epithelialization at time of separation, need for additional excision and grafting, and hypertrophic scaring. Nine patients (9/133 = 6.8%) experienced premature separation attributed to several different etiologies. One patient experienced separation of EZ Derm® on postoperative day 4 that was applied to the shoulder. Another patient had separation from the back on POD 2. Both of these failures were attributed to poor positioning (2/133 = 1.5%). The first patient noted was not put in a splint to limit shoulder mobility and the second patient likely had excessive frictional forces due to normal repositioning while lying in bed. These patients went on to have fully healed wounds with local wound care alone.

Clinically significant signs of infection were reported in 4 patients (4/133 = 3.0%). In 3 of these patients, cellulitis and/or gross purulence was noted with premature separation of the xenodressing on PODs 7, 9, and 3, respectively. All of these patients had complete wound healing with local wound care. One patient had cellulitis as well as inadequate wound debridement that led to premature separation on POD 3. This patient required antibiotic therapy as well as excision and grafting. Similarly, 2 other patients had premature separation on POD 4 and 5, but without clinical signs of infection. Both of these patients also required subsequent excision and grafting as these wounds were deemed unlikely to heal spontaneously within 3 weeks. Lastly, another patient had premature separation of EZ Derm® from the chest. The etiology of this failure remained indeterminate as there were no documented signs of infection or positioning issues.

Three additional patients required excision and grafting; however, they did not present with premature separation. Spontaneous separation was noted on PODs 15, 14, and 13, respectively. Upon evaluation in clinic, these patients were noted to have healed partial-thickness wounds around small areas of full or deep partial-thickness wounds. These wounds were not likely to fully heal by 3 weeks and thus required excision and grafting to achieve timely closure. Overall, 6 patients were noted to require subsequent excision and grafting to achieve complete wound healing (6/133 = 4.5%), generally due to unintentional misevaluation of initial wound depth before EZ Derm® application.

Three patients were noted to have prolonged adherence and/or incomplete epithelialization at the time of EZ Derm® separation on PODs 11, 13, 11, respectively (3/133 = 2.3%). In these patients, residual wounds were deemed likely to fully heal by 3 weeks after burn, and accordingly, local wound care was initiated. All 3 patients ultimately achieved fully healed wounds.

Lastly, 2 patients were noted to have hypertrophic scaring on PODs 69 and 91. One of these patients had a 33% TBSA partial-thickness burn treated only with EZ Derm® (6060 cm^2^) and was noted to have 2 small areas of hypertrophic scaring, each less than 2 cm^2^. These lesions were subsequently successfully treated with steroid injections. The second patient was noted to have hypertrophic scaring on bilateral upper extremities. This patient also had prolonged adherence of EZ Derm® requiring removal in clinic that revealed healthy granulating tissue. These wounds were subsequently grafted. Our records did not indicate if areas of hypertrophic scaring were associated with split thickness skin graft (STSG) or partial-thickness areas successfully treated with EZ Derm® alone. Pressure garment therapy was continued in this patient without further progression of hypertrophic scaring. Because of the delayed onset of hypertrophic scaring, only patients with more than 60 days of follow-up were considered to have been observed long enough for hypertrophic scaring assessment, as such, the overall rate of hypertrophic scaring was determined to be 3.3% (2/60).

In addition to its use on partial-thickness burns, EZ Derm® was used to cover STSG donor sites in 5 patients (5/154 = 3.2%). No data on time to heal was accurately recorded. All 5 patients went on to have complete healing without complication. Overall time to complete EZ Derm® separation was not recorded in this study. Most patients were seen in the outpatient clinic, at which time either the EZ Derm® had been removed prior to the visit or during the visit. Because daily inspection and timely removal was not possible, accurate measure of this metric was not possible. However, it is our experience that EZ Derm® separates from partial-thickness wounds within 2 weeks, which is in agreement with prior studies that looked more rigorously at this metric.^6^^,^^10^ Firm adherence after this period typically implies a deeper burn.

## DISCUSSION

The early history of xenograft use as a human skin substitute dates back to Canaday's early experiments with lizard skin in 1682, and later with Reverdin and Lee's in the late 1800s with bovine and porcine dermis, respectively.^11^^-^13 These early trials were aimed at permanent skin replacement and predictably failed because of graft rejection. In 1881, Girdner^14^ took another step forward with the use of fresh cadaveric skin to cover wounds. While these early pioneers were met with failure, they laid the foundation for those who followed. It was not until Brown's work in 1953 when the use of cadaveric skin became routine in the management of burn care.^15^ Despite limited availability early on, benefits noted with the use of allograft included decreases in infection, fluid and heat loss, and pain, as well as excellent wound bed preparation prior to application of autograft.^16^ The benefits observed in wounds treated temporarily with allograft rekindled interest in xenografts as temporary dressings.

Porcine products are currently the most widely available xenographic biologic dressing in burn care.^5^ Porcine skin has long been favored over other species as a viable substitute for allograft in part because of its similarities to human skin, such as comparable epidermal thickness, epithelial turnover and migration, hair distribution, dermal elastin structure, and dermal collagen macrostructure.^17^^,^^18^ Interestingly, pigs are the only other animal susceptible to sunburn in a manner similar to humans.^17^ Differences include lack of stratum lucidum, lower elastic content, and tighter, more densely packed collagen bundles.^17^^,^^18^

There are currently many different porcine-derived products from a variety of manufacturers in the market. Current preparations include fresh, fresh frozen, lyophilized, irradiated, and aldehyde cross-linked.^19^^-^23 In addition to the fresh frozen preparations, lyophilized and irradiated forms require refrigeration to prolong shelf life. The lyophilized and irradiated forms have the added benefit of sterility that is a common concern when using fresh and fresh frozen preparations. Fresh, fresh frozen, lyophilized, and irradiated forms are also similar in that they are all composed of dermal and epidermal layers. Aldehyde cross-linked preparations differ from these in that the processing essentially removes all epidermis and dermal appendages, leaving a sterile acellular dermal matrix that can be used either side down and can be stored at room temperature. EZ Derm® is one such example with a shelf life of 18 months.^24^^,^^25^

EZ Derm® has been used at our institution over the last 4 years for the treatment of partial-thickness burns. During this time, 157 patients were treated, and 84.7% (133/157) were followed until complete wound healing had occurred. There were 18 complications found in 16 patients, resulting in an overall complication rate of 12% (16/133). Out of these complications, 50% (9/18) presented with premature slough of EZ Derm® as the present sign of poor graft positioning (1.5%, 2/133), infection (3%, 4/133), inadequate debridement (2.2%, 3/133), and indeterminate reasons (0.8%, 1/133) (average = 4.3 days, STD = 2.3 days, Median = 4 days).

Most of the beneficial effects of EZ Derm® in burn patients appear to result from its tight adherence to the wound bed.^26^^,^^27^ These include reduction in pain, fluid loss, and infection, providing a moist wound environment, and an inherent hemostatic property.^23^^,^^27^ Accordingly, loss of this adherence, by any mechanism, hinders wound healing. Wound bed adherence has been shown to be primarily the result of the fibrin-collagen bond.^26^ For all types of porcine dressings, this bond serves as the primary wound adherence mechanism since subsequent “take” or graft vascularization does not occur.^5^^,^^18^,^28^^-^33 This represents a fundamental difference between porcine products and allograft, which exhibits vascularization within 3 to 5 days. The etiology of this difference has yet to be determined, but current theories point toward differences in dermal collagen ultrastructure, and more specifically the tighter packing of collagen bundles found in porcine dermis.^17^^,^^34^ The overall result is the inability of porcine dermis to be invaded by human fibroblast, endothelial cells, or neutrophils.^18^^,^^31^^,^^35^ This quality is of benefit when used as a temporary dressing because there is no risk of zoonosis and the dressing may be left in place until it spontaneously separates.^28^^,^^36^ It should be noted, however, that because porcine products do not undergo vascularization, they are not technically xenografts, but instead a biologic dressing or xenodressing.^5^

In the present study, 16.8% (3/18) of complications were found to have significant areas of incomplete epithelialization at the time of EZ Derm® removal, and 11% (2/18) presented with hypertrophic scaring (3.3% = 2/60). While porcine dermis may not “take,” it has been found to have a positive impact on human keratinocyte proliferation and differentiation, as well on fibroblast proliferation.^22^^,^^35^ Recent work by Zajicek et al has shown that the collagen structure in porcine dermis promotes normal keratinocyte differentiation and stratification. The authors conclude that this is largely responsible for the healing effects of porcine xenodressing on partial-thickness burns.^22^ Increased effect on proliferation of fibroblast and endothelial cells are also thought to help in would bed preparation when used on excised, full-thickness wounds.^35^ These effects are likely responsible in part for the healing effects of EZ Derm®; however, it is unknown if these properties are the cause of hypertrophic scaring. In this series, both cases of hypertrophic scaring were more likely the result of insufficient debridement and overall delay in complete wound closure than a result of an intrinsic property of EZ Derm®.

Clinically significant infections were seen in 22% (4/18) of complications and 3% of patients overall (4/133). These patients presented with cellulitis and/or gross purulence. In all cases, the presenting sign of infection was nonadherence to the wound bed. Strong adherence is thought to be responsible for decreased rates of infection seen with porcine xenodressings, including EZ Derm®. While these dressing do not have any inherent antibacterial properties, it is thought that their firm adherence augments host immunity and provides a bacterial barrier.^3^^,^^30^^,^^37^ The exact mechanism for this has yet to be elucidated, but it is thought that wound adherence prevents the accumulation of dying leukocytes in the wound bed which may have an antiphagocytic effect, thus inhibiting bacterial clearance.^37^

Wound adherence also reduces evaporative losses. In an early study by Salisbury et al, evaporative losses from allograft were compared to fresh porcine full-thickness skin. He found that allograft was superior, but that the porcine alternative also greatly reduced evaporative losses.^38^ While EZ Derm® was not used in this study, it is likely that it would not be as effective as allograft or fresh xenodressing because of the lack of an epidermal component, which serves as the major vapor barrier. However, the clinical significance of this phenomenon is not known, as studies of EZ Derm® use in patients with toxic epidermal necrolysis demonstrate decreased evaporative losses and fluid requirements.^39^ Evaporative losses and fluid requirements were not evaluated in the present study.

More recent work on the benefits of xenodressings in burn treatment has shown that they prevent further burn wound progression on fresh burns.^19^ By reducing the local inflammatory response in fresh burns, inhibition of burn wound progression, and even reversal of ischemia in tissue in the zone of stasis, has been observed. Beyond the beneficial effect observed on wounds, porcine xenodressings have also been shown to reduce systemic inflammatory response syndrome, resulting in normalization of temperature and vital signs when compared to Betadine ointment–covered gauze.^3^^,^^19^

Early concerns about the use of porcine dressings included the occurrence of immunogenicity and rejection on burn wounds. Studies on fresh porcine skin found a type II humoral immune response that would typically lead to rejection within 12 days.^6^^,^^40^ More recent studies suggest the Gal epitope is responsible for this immune response.^41^ Because of the lack of cellular invasion, type IV hypersensitivity reactions are not observed.^40^ Problems with rejection have only been reported with early usage of fresh and fresh frozen preparation in which a patient was continuously covered while awaiting repeat harvest of autograft.^42^^,^^43^ In these cases, it was noted that the dressing would eventually fail to remain adherent after application. However, similar rejection has not been reported when used on partial-thickness burns, likely due to the self limited nature of its use in which host epidermis reconstitutes under the dressing.^44^ In an effort to eliminate the humoral response, porcine dermis was later cross-linked with aldehyde.^33^ This served to prevent any measurable host response, as well as improving the handling and characteristic of the dressing.^6^ No report of rejection was noted in this study; however, because of the retrospective nature of this study, the possibility of unrecognized early rejection cannot be excluded, although unlikely, given the known immunologic effect of collagen products treated with aldehyde.^45^

Because of aldehyde treatment, EZ Derm® may be stored at room temperature for up to 18 months. Room temperature storage capabilities result from the elimination of residual enzymes that remain in other preparations and require refrigeration to suppress their activity and subsequent product degradation.^6^ Aldehyde processing has not been found to substantially change the dermal structure.^6^ Overall, the clinical significance of humoral rejection has not been established, but it is likely not significant given the overall benefits observed from application of these dressings.

Xenodressings also have the added benefit of inherent hemostatic properties resulting from the activation of the coagulation cascade by contact with collagen. This is particularly beneficial in burn care as application of xenodressings over freshly excised wounds can help to limit overall blood loss. A study by El-Khatib et al^8^ demonstrated this benefit over a comparable synthetic analog, Biobrane, in the treatment of freshly excised burns. In the present case series, no clinically significant hematomas were reported.

One of the major benefits of porcine xenodressings is pain reduction. While this metric was not specifically investigated in the present study, a wealth of past studies support the analgesic effect of temporary wound closure achieved with porcine xenodressings.^8^^,^^21^^,^^32^^,^^39^^,^^42^^,^^44^,^46^^-^50 It is our experience that application of EZ Derm® results in significant baseline pain reduction, as well as elimination of pain associated with daily dressing changes.

Use of porcine dressing has also been associated with decrease in Length of Stay (LOS) and overall cost. In a study by Still et al,^51^ patients with partial-thickness burns were treated with xenodressing and discharged. Patients with toxic epidermal necrolysis and those with inhalation injury were excluded in this study. Length of stay was reported as 7 days on average with 19.3% needing readmission for subsequent excision and grafting for patient with up to 25% TBSA. They found that application of porcine xenograft resulted in overall decreased LOS even when accounting for these additional procedures.^51^^-^53 In the present series, 4.5% (6/133) of patients with up to 25% TBSA, excluding inhalation injuries, were found to require excision and grafting after EZ Derm® placement with an average LOS of 8 days.

The possibility of disease transmission is an important consideration with products derived from allogenic of xenogenic sources. Testing for transmissible diseases contributes a large part to the overall expense of allograft. Concerns about sterility in fresh and fresh frozen products eventually lead to the manufacturing of lyophilized and irradiated products, and finally to aldehyde cross-linked product.^25^ To date, there have been no reported cases of split-thickness porcine xenografts being responsible for disease transmission.^7^^,^^25^

The use of EZ Derm® has few absolute contraindications. One such indication often sited in the literature is the possible resistance to use on patients of the Muslim faith due to their aversion to porcine produces.^21^^,^^54^ However, it is our experience with interactions involving local religious leaders that this aversion is limited to ingestion of porcine products and does not apply to topical application such as with EZ Derm®. Lastly, a history of porcine allergy is viewed as a reason to avoid use, although no report of an allergic reaction could be found in the literature in regard to EZ Derm®. An allergic reaction is far more likely in the setting of fresh and fresh frozen porcine xenografts, because of the preparation methods of EZ Derm® as described earlier.

We did not utilize EZ Derm® on pediatric patients or hand burns because of concerns of graft stability and shear in areas or patients who required early mobility or could not comply with immobility/limited mobility instructions. Experience in use in the head and neck was limited by mobility/shear concerns as well.

Another measure of utility of burn wound care is cost. Cost of wound care is determined not only by material cost but also by related expenses of cover dressing and the need for nursing expertise. A cost comparison for patients treated similarly with respect to in-hospital therapies, between EZ Derm® and daily topical antimicrobial dressings, for a similar size wound, indicates a significant cost difference. The price for EZ Derm® at our institution is US $140.00 for a 7 cm × 8 cm sheet. Daily topical antimicrobial dressing changes as an outpatient, utilizing home health care, are more than $150.00 per day.

Wounds treated with EZ Derm® require no specific outpatient treatment or home health care interventions. It would appear that EZ Derm® application and utilization is quite cost-effective.

The strength of this study comes from the large number of consecutive patients and length of follow-up. Our study is limited by the retrospective nature of the data collection.

## CONCLUSIONS

EZ Derm® has played a significant role in the management of partial-thickness burns at our institution over the past 4 years. During this time, it has proven to be a robust wound dressing that provides well-tolerated wound coverage with minimal complications. Wound healing and wound bed preparation outcomes are favorable. The utilization of EZ Derm® appears to be cost-effective as well. We recommend its use in cases of partial-thickness burns as described.
